# Resection of pancreatic metastatic renal cell carcinoma: experience and long-term survival outcome from a large center in China

**DOI:** 10.1007/s10147-019-01399-w

**Published:** 2019-03-07

**Authors:** Yang Ma, Jingrui Yang, Kai Qin, Yiran Zhou, Xiayang Ying, Fei Yuan, Minmin Shi, Jiabin Jin, Di Wang, Jiangning Gu, Dongfeng Cheng

**Affiliations:** 1 Department of General Surgery, Ruijin Hospital, School of Medicine, Shanghai Jiao Tong University, No.197 Ruijin Number Two Road, Huangpu District, Shanghai, 200025 People’s Republic of China; 2 Department of Pathology, Ruijin Hospital, School of Medicine, Shanghai Jiao Tong University, No.197 Ruijin Number Two Road, Huangpu District, Shanghai, 200025 People’s Republic of China; 3 Research Institute of Digestive Surgery, Ruijin Hospital, School of Medicine, Shanghai Jiao Tong University, No.197 Ruijin Number Two Road, Huangpu District, Shanghai, 200025 People’s Republic of China; 4 Department of Scientific Research, Eyes and ENT Hospital of Fudan University, No. 83 Fenyang Road, Xuhui District, Shanghai, 200025 People’s Republic of China; 5 Present Address: Department of Hepatobiliary Surgery, The First Affiliated Hospital of Dalian Medical University, No. 222 Zhongshan Road, Xigang District, Dalian, 116011 Liaoning People’s Republic of China

**Keywords:** Metastatic renal cell carcinoma, Pancreatic metastasectomy, Surgical resection

## Abstract

**Purpose:**

This study aimed to determine the outcome of pancreatic metastatic renal cell carcinoma (PmRCC) after treatment and share the relevent results.

**Methods:**

In total, 13 patients with PmRCC were diagnosed and treated in our institution from December 2013 to October 2017. We retrospectively reviewed the records and analyzed the patient demographics, perioperative outcomes, and overall survival. Simultaneously, our experience including treatment and misdiagnosis was shared.

**Results:**

The median time between nephrectomy and reoperation for pancreatic recurrence was 11 years (range 1–20 years). Four patients had multiple tumors and nine patients had solitary tumor. Five patients accepted distal pancreatectomy, and five patients underwent pancreaticoduodenectomy. One patient underwent total pancreatectomy, one patient underwent duodenum-preserving pancreatic head resection plus distal pancreatectomy, and one patient underwent exploratory laparotomy and gastrointestinal bypass due to widespread metastasis with clear obstructive symptoms. The misdiagnosis rate of preoperative diagnosis at our center was 69.2% (9/13). The median follow-up duration was 26 months (range 7–53 months, until June 2018). By the end of follow-up, 12 patients were alive and one patient died of gastrointestinal bleeding within 1 month after surgery.

**Conclusions:**

PmRCCs are uncommon, but pancreatic metastasectomy has a relatively good prognosis and may, therefore, be a good therapeutic choice for patients with PmRCCs. Because PmRCC occurs long after the primary tumor resection, long-term follow-up is necessary. Besides, detailed medical history and specific manifestation in imaging features could contribute to avoiding misdiagnosis.

## Introduction

The prevalence of renal cell carcinoma (RCC) is an increasing cause of mortality in USA and has accounted for 14,970 deaths in 2018 [1]. Metastasis from RCCs is common, and in approximately 25% of patients, metastasis is already present during RCC diagnosis [2]. RCC primarily metastasizes to the lung, liver, bone, and adrenal tissue [3]. Metastatic lesions to the pancreas are relatively uncommon (< 10%) and account for only 2% of all pancreatic neoplasms [4]; thus the misdiagnosis rate is relatively high.

The reported incidence of recurrence after nephrectomy is 20–30%, and the median relapse interval is 15–18 months; 85% of recurrences occur within 3 years of nephrectomy [5, 6]. However, PmRCC often occurs a long time after nephrectomy, which makes them difficult to detect [7–10]. Usually, patients with PmRCC choose the Departments of General/Pancreatic Surgery as the First Clinical Department. Therefore, it is difficult to differentiate PmRCC from pancreatic neuroendocrine tumor (PNET), pancreatic ductal adenocarcinoma, or other primary pancreatic tumors, as each of them has different therapeutic modalities with different prognoses.

Targeted therapy is recommend as the first-line treatment for recurrent RCC [11, 12]. A few studies reported that tyrosine kinase inhibitor-based chemotherapy showed favorable overall survival in patients with recurrent RCC [13]. In addition, cytokines such as interleukin-2 and interferon-α were effective in the treatment of metastatic RCC [14]. However, studies on surgery for PmRCC are presently limited in the literature. Therefore, this study aimed to describe 13 cases of PmRCC, which may be one of the largest cohorts thus far, with detailed follow-up information from over 10 years at our center. We have not only summarized our experience of the diagnosis and treatment of PmRCC but also presented the lessons learned from our mistakes to share our experience of surgical therapy of PmRCC.

## Patients and methods

In total, 13 patients underwent treatment for PmRCC from December 2013 to October 2017 at our center, and all the diagnoses were validated by histopathology. We retrospectively analyzed patient demographics, preoperative details, imaging data, medical history, treatment modalities including the type of surgery, surgical outcomes, and long-term survival. The diagnostic criteria and classification for PNET were based on imaging features and guideline of European Neuroendocrine Tumor Society (ENETS) [15]. For the metastasis of RCC, the detection and diagnosis mainly depend on the medical history and pathology results. Finally, we summarized our experience with some representative images and presented lessons from our daily clinical practice.

Preoperative examination included physical examination, computed tomography (CT), abdominal ultrasound, endoscopic ultrasound, magnetic resonance imaging (MRI), and endoscopic retrograde cholangiopancreatography, as required.

Before surgery, the advantages, disadvantages, and risks of operations were explained to the patients and/or their relatives. The surgical procedures were selected according the standard clinical process of our center. Each patient was assessed by a multidisciplinary clinical team. Perioperative mortality was defined as death within 30 days of operation. Follow-up information was obtained by a review of the hospital records and direct patient communication. Complete survival information was available for all 13 patients. The endpoint of follow-up was June 1, 2018. The primary endpoint was overall survival.

All the imaging data were collected from the Department of Radiology, Ruijin Hospital, with the consent from the patients or their relatives. All patient data were processed to maintain anonymity. This study was approved by the Ethics Committee of Ruijin Hospital, which is affiliated to Shanghai Jiao Tong University, and all the participants signed an informed consent form.

## Results

### Patient demographics

The average age of the patients was 64.7 years (range 54–85 years; median, 62 years). Eight patients underwent right nephrectomy, and five patients underwent left nephrectomy. All the patients were pathologically diagnosed with RCC during the primary operation. None of the patients underwent chemotherapy or radiotherapy after nephrectomy. The median size of the tumors was 44 mm (range 11–75 mm). The median time between nephrectomy and reoperation for pancreatic recurrence was 11 years (range 1–20 years). Patients accepted follow-up in every 3–6 months for 3 years, then annually for up to 5 years after primary nephrectomy. The follow-up examinations include history and physical examination (H&P), blood tests, and abdominal imaging (with enhanced CT or MRI). However, none of the patients attended regular follow-up after 5 years since primary nephrectomy. Six patients were symptomatic when diagnosed with pancreatic metastases: four of them had abdominal pain and two had weight loss. The remaining patients had no symptoms, and recurrence was detected by routine examination (Table 1).

**Table 1:** Demographic and clinical characteristics

Case	Age/sex	Symptoms	Operative procedure	Location/number	Size (mm)	Preoperative diagnosis	Pathological type of primary tumor	Side of the primary tumor	Time since primary operation (years)
1	62/F	Routine examination	DP	Tail/1	75	PNET	RCC	Right	15
2	55/M	Abdominal pain	PD	Head/1	63	PNET	RCC	Left	6
3	54/F	Routine examination	DP	Body and tail/2	25	PNET	RCC	Right	1
4	59/F	Abdominal pain	PD	Head/1	44	Pancreatic RCC	RCC	Right	11
5	68/F	Abdominal pain	DPPHR + DP	Head, body, and tail/3	30	Pancreatic RCC	RCC	Left	10
6	65/F	Routine examination	PD	Head/1	18	PNET	RCC	Left	10
7	85/M	Routine examination	DP	Body/1	54	PNET	RCC	Right	20
8	62/F	Routine examination	DP	Body/1	11	PNET	RCC	Left	11
9	56/F	Abdominal pain	PD	Head, body, and tail/3	47	PNET	RCC	Right	10
10	61/M	Weight loss	PD	Head/1	50	Pancreatic RCC	RCC	Left	7
11	77/F	Routine examination	Total pancreatectomy	Head, body, and tail/3	34	Pancreatic RCC	RCC	Right	20
12	70/M	Routine examination	DP	Body/1	28	PNET	RCC	Right	14
13	67/M	Weight loss	Exploratory laparotomy	Head/1	72	PNET	RCC	Right	20

*DP* distal pancreatectomy, *PD* pancreaticoduodenectomy, *DPPHR* duodenum-preserving pancreatic head resection, *RCC* renal cell carcinoma, *PNET* pancreatic neuroendocrine tumor

### Differential diagnosis and lessons

**Fig. 1:**
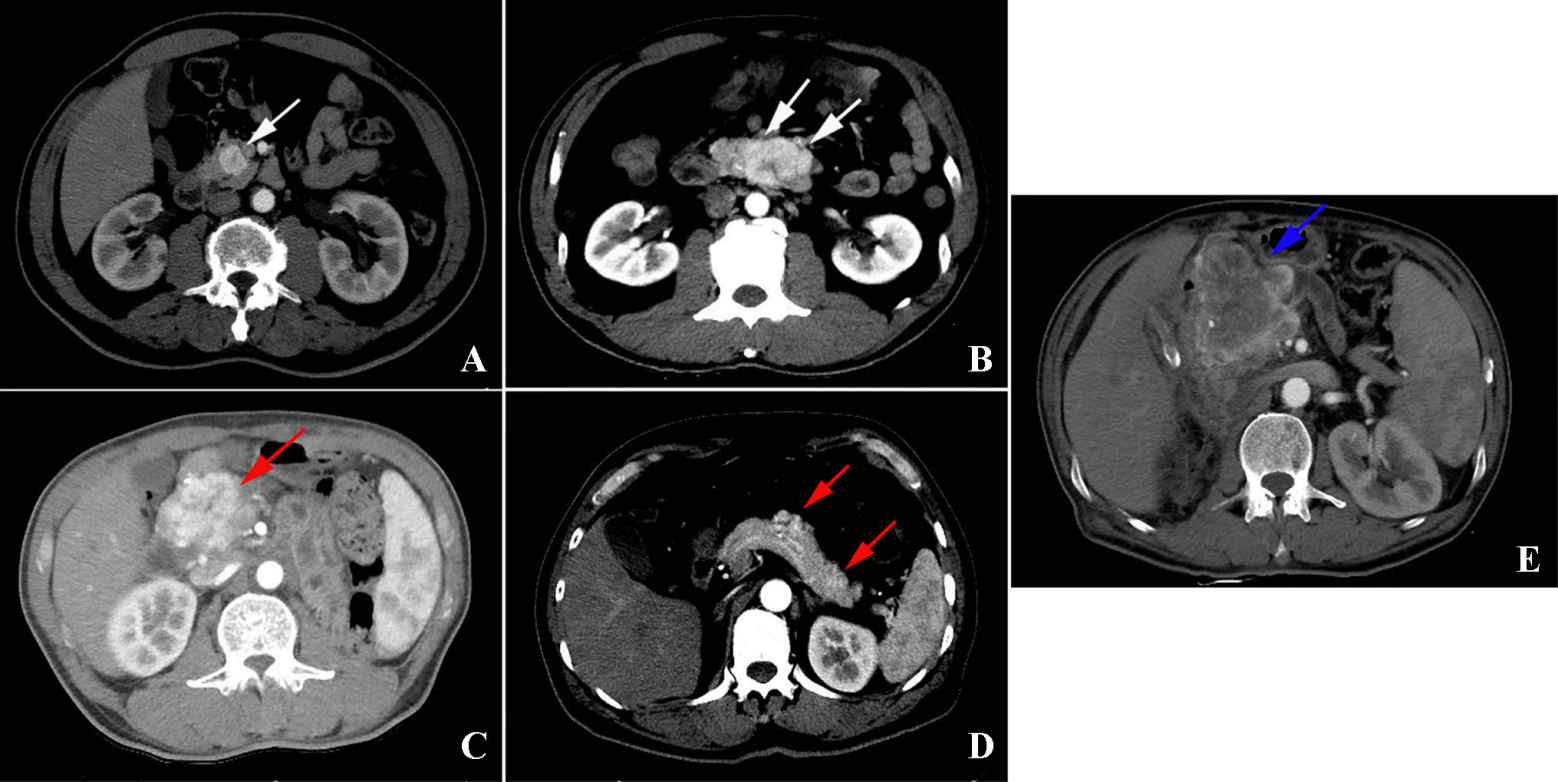
**a** Solitary pancreatic endocrine tumor in the pancreas. **b** Multiple pancreatic endocrine tumors in the pancreas. **c** Solitary pancreatic renal cell carcinoma metastases in the pancreas. **d** Multiple pancreatic renal cell carcinoma metastases in the pancreas. **e** A patient with pancreatic renal cell carcinoma metastases misdiagnosed with a pancreatic endocrine tumor

PmRCC is difficult to diagnose because of the following reasons: First, PmRCC is very rare, and it is difficult to differentiate between the results of enhanced CT for PmRCC and those for PNET. This is especially the case for nonfunctional PNET, as it often appears as a hypervascular image on CT (Fig. 1a–d). Second, although patients have a history of malignancy, a time to recurrence of more than 5 years is considered a clinical cure, which makes it difficult for general surgeons to suspect PmRCC. Finally, renal metastasis to the pancreas is less prevalent than that to other organs such as the liver and lung.

According to the preoperative examination, multiple lesions were present in four patients; the remaining patients had solitary lesions (detected by CT or MRI, the number of metastasis was listed in Table 1). Preoperatively, seven patients were diagnosed with PNET (two patients were considered to have reached G3), and two patients were diagnosed with pancreatic cancer. The misdiagnosis rate was 69.2% (9/13). In four patients, RCC metastasis and endocrine tumors could not be excluded (Table 1).

**Fig. 2:**
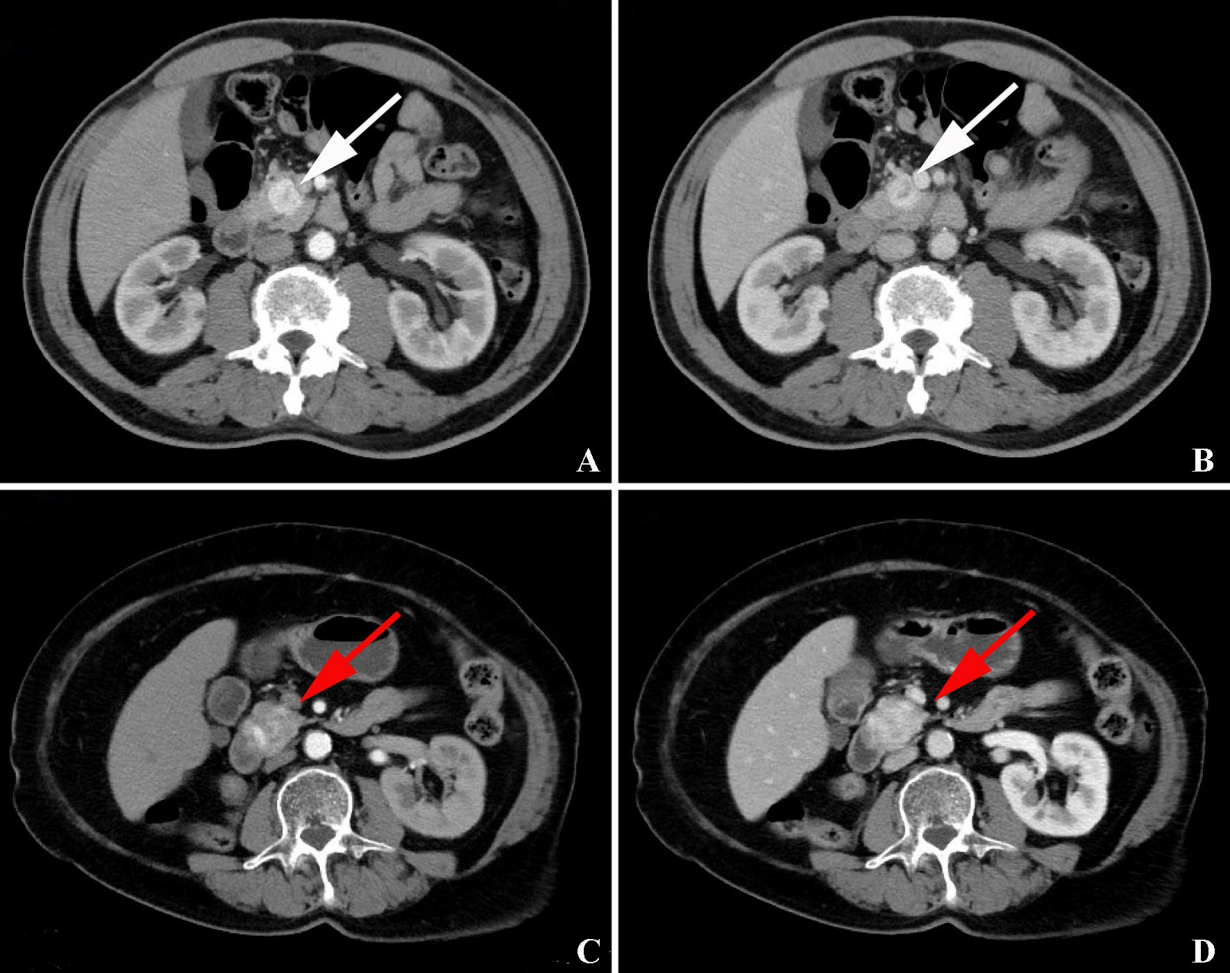
**a** The representative arterial phase figure of pancreatic endocrine tumor in the pancreas. **b** The representative venous phase figure of pancreatic endocrine tumor in the pancreas. **c** The representative arterial phase figure of pancreatic renal cell carcinoma metastases in the pancreas. **d** The representative venous phase figure of pancreatic renal cell carcinoma metastases in the pancreas

According to our treatment experience of the 13 cases of RCC, our center summed up some directions which may help doctors to differentiate the diagnoses. First, some biochemical markers such as chromogranin A (CgA) and neuron-specific enolase (NSE) maybe useful diagnostic biomarker for neuroendocrine tumor [16, 17]. Which is accordance with the perspective of Raoof et al. that CgA level could be helpful to predict biologic behavior of small nonfunctional PNET [18]. Second, PNET is often characterized by hypervascularity and is more conspicuous on earlier phases of enhancement in the enhanced CT [19]. However, for the metastasis of RCC, the enhancement usually appears in venous phase and balance phase, which reminds surgeons to carefully identify the difference in imaging features (Fig. 2a–d). Additionally, according to a recent study, relative percentage washout (RPW) in CT is helpful for differentiating metastasis of RCC from PNET [20].

### Therapeutic modalities

As shown in Table 1, mainly based on tumor location, five patients underwent distal pancreatectomy (DP) and five patients underwent pancreaticoduodenectomy (PD). One patient underwent total pancreatectomy and another patient underwent duodenum-preserving pancreatic head resection plus DP for the multiple metastatic lesions. One 67-year-old patient was unable to undergo radical surgery due to a large mass that metastasized to the pancreatic head and invaded the duodenum and great vessels such as the superior mesenteric artery/superior mesenteric vein with obstructive symptoms; this patient finally underwent only gastrointestinal bypass.

The median blood loss was 200 mL (range 100–600 mL). The median operation time was 210 min (range 80–330 min), and the median postoperative hospital stay was 16 days (range 8–45 days). Two patients experienced obvious postoperative complications: one patient suffered from pancreatic fistula, was sent to the intensive care unit, and required a longer recovery time than all the other patients; the other patient died 29 days after surgery because of acute gastrointestinal bleeding and hemorrhagic stroke. All the patients who underwent pancreatic resection in our study had negative margin according to intraoperative frozen section and postoperatively pathological diagnosis. The median follow-up duration was 26 months (range 7–53 months, until June 2018). One patient who underwent bypass surgery died of gastrointestinal bleeding within 1 month after surgery. The remaining 12 patients were alive without recurrence until the last follow-up (Table 2).

**Table 2:** Perioperative details, follow-up, and status

Case	Blood loss (mL)	Operation time (min)	Postoperative hospital stay (days)	Complications	Follow-up (months)	Status
1	100	100	10	–	53	Alive
2	300	240	17	–	44	Alive
3	250	120	8	–	36	Alive
4	200	210	19	–	30	Alive
5	500	240	16	–	30	Alive
6	600	330	12	–	28	Alive
7	200	120	13	–	22	Alive
8	100	180	17	–	18	Alive
9	350	260	41	Pancreatic fistula (grade B)	14	Alive
10	400	240	45	Postoperative hemorrhage and stroke	7	Alive
11	200	300	22	–	10	Alive
12	200	90	13	–	10	Alive
13	100	80	12	–	/	Dead

Of note, one patient accepted Sandostatin (Novartis Pharma Schweiz AG, Switzerland) treatment in the perioperative period because he was misdiagnosed with PNET. This patient was considered to have PNET according to the imaging results and intraoperative frozen detection. However, owing to the poor outcome and final immunohistochemistry results, we diagnosed him with PmRCC. This case reiterates that PmRCC should be considered in patients with pancreatic tumors who have a medical history of RCC, even after long-term survival, as this approach may relieve pain and the economic burden (Fig. 1e).

## Discussion

Although our center is one of the largest pancreatic centers in the world, with more than 800 pancreatectomies performed per year, PmRCC still remains a rare tumor, the misdiagnosis rate of which was nearly 70% even in patients who underwent preoperative multidisciplinary assessment. Thus, experience and lesson summarization are important for the community.

PmRCC appears to have the following characteristics: First, a long period between initial nephrectomy and the diagnosis of metastatic lesions. At our center, the median interval was 11 years (range 1–20 years), which is even longer compared to a median interval of 6 years (range 5–30 years) reported in previous studies [5, 11]. Therefore, patients with a medical history of nephrectomy should undergo periodic follow-up for more than 10 years after surgery to detect recurrence [21]; consequently, a 5-year follow-up is not sufficient for such patients. Second, pancreatic metastatic lesions are usually asymptomatic and are accompanied by common symptoms of abdominal pain and weight loss, which are often overlooked by patients [22]. Third, the outcome of PmRCC resection is relatively favorable over that of primary pancreatic tumors such as pancreatic adenocarcinoma which probably was attributed to its tumor biology. In our case series, the median follow-up after pancreatic resection was 26 months (range 7–53 months). During this period, none of the patients died from recurrence. Finally, the pancreas appears to be an isolated site of RCC, and synchronous metastases were a rare phenomenon (0–29% in the literature) [2, 7], which is consistent with our results (0%).

Identifying pancreatic metastasis is a challenge because metastatic lesions to the pancreas are extremely rare and attribute to only 1–2% of all pancreatectomies performed in some institutions [11, 22]. At present, PET-/CT, MRI, and even endoscopic ultrasound are used as routine examinations for the detection of pancreatic metastases. The typical manifestations of pancreatic metastases on CT were well-defined margins and greater enhancement than that seen for a normal pancreas [11]. However, the hypervascular appearance of PmRCC often shares the same morphological and imaging features with PNETs on CT [11], which lead to the high misdiagnosis rate. At our center, nine (69.2%) patients were diagnosed with PNET before surgery, of which only four (30.8%) were correctly diagnosed. Endoscopic ultrasound-guided fine-needle aspiration is an effective method to identify the histologic type and origin of pancreatic metastases [23], but the risk of tumor seeding must be considered and the technique was also a challenge with the possibility of false negative.

Surgical resection is probably the best choice for PmRCC treatment according to our results and other published ones, although metastasectomy is usually not recommended for most other cancers [13, 14, 24]. The 5-year survival rate of patients with untreated metastatic RCC was 13% compared to 65% after surgical resection [25]. A retrospective study evaluating patients who underwent non-operative management of PmRCC showed a 5-year survival rate of 47% compared to 88% in the operative group, which indicated that pancreatic metastases from RCC were favorable for resection, even in the presence of another metastatic site or multifocal pancreatic disease [26]. At our center, the perioperative morbidity and mortality were 15.4% and 7.7%, respectively, which were not worse than the previously reported values of 47% and 6.4%, respectively [25, 27]. Therefore, careful patient selection and perioperative management should be emphasized because it may be difficult to achieve ideal therapeutic effect in patients with highly advanced disease.

Patients who undergo surgical treatment for pancreatic metastases of RCC could acquire a favorable long-term survival [28, 29], with 3-, 5-, and 10-year overall survival rates of 72%, 63%, and 32%, respectively [22]. Konstantinidis et al. reported a 5-year actuarial survival rate of 61% and a better median survival (8.7 years) than that for other types of metastases [30]. At our center, all the patients were alive without recurrence at the last follow-up period, which indicated the effectiveness of improving survival. It is worth noting that one patient underwent total pancreatectomy due to the multiple lesions. For this patient, we used long-acting insulin at the dose of 8 units twice a day and oral hypoglycemic agent to control postoperative blood glucose; we also advised patient to take oral trypsin tablets if necessary. In the postoperative follow-up, the blood glucose is effectively controlled and the patient recovered well with acceptable QOL. This example illustrates that by closely monitoring blood glucose and providing extra trypsin after total pancreatectomy, the patient could have a better quality of life. In addition to the widely used prognostic factor model from the Memorial Sloan Kettering Cancer Center [31–33], vascular invasion [2], presence of symptoms [34], and tumor size more than 3 cm [35] may be associated with an increased risk of death. However, the localization and number of metastatic lesions have no effect on the site of metastases [34], which is in line with our current results. Although a few researchers believe that lymphadenectomy should be considered for pancreatic metastasis [35], there is no convincing evidence to indicate that the involvement of lymph nodes in pancreatic metastasis may affect prognosis [8, 36, 37].

The pancreas is the common site to which RCC metastasizes, and such metastasis typically occurs a long time after nephrectomy [21, 38]. Both hematogenous spread and lymphatogenous spread were considered to be the underlying mechanisms of RCC metastasis to the pancreas [2]. In a previous study, no relation was found between the side of the primary kidney tumor and the site of the pancreatic metastasis, which indicated that local (lymphatic) spread may not be the route of metastasis [6, 29]. In addition, lymph-node positivity was rarely observed during surgery [8, 29, 39]. However, the discrepancy between the relative frequency of pancreatic metastases and the absence of metastases to other organs could not be explained by systemic spread [6, 8]. Therefore, further research on the biochemical mechanisms underlying tumor metastasis has been conducted at our center.

Our study has a few limitations. First, although surgical resection might be an ideal option for patients with PmRCC, long-term efficacy of surgery requires further study. Second, it is difficult to avoid selection bias in this retrospective study because of the preferences of the surgeons and patients. Finally, the small sample size at our center does not allow further statistical analysis. Longer follow-up period and larger cohort should be further investigated, since current studies of PmRCC are just at the beginning (Table 3).

**Table 3:** Recent studies on the pancreatic resection of metastatic renal cell carcinoma

Name	*n*	Age (years)	Size (mm)	Complications	Time since primary operation (years)	Follow-up (months)	Median survival (months)	5 years survival (%)
Yagi et al. [38]	9	66 (52–83)	28 (10–39)	3	11.5 (0–19)	23.5 (3–138)	/	/
Schwarz et al. [25]	62	54 (31–75)	35 (10–250)	4	10 (0–25)	91 (12–250)	52.6	63
Konstantinidis et al. [30]	20	68.5 (44–84)	30 (8–120)	/	8.7 (0–22)	36.8 (0.5–143)	104	61
Tosoian et al. [2]	42	66.4 (32–87)	38 (8–105)	26	11.2 (0–28)	84 (1–278)	66	51.8
Schauer et al. [5]	10	62 (54–73)	/	2	9 (0.4–23)	56 (2–77)	30	/

## Conclusion

PmRCCs usually progress slowly and are often confused with PNET. Therefore, close and long-term follow-up (more than 10 years) is necessary for such cases. Once a hypervascular pancreatic lesion is detected in the follow-up period, it is essential to review the medical history of the patient and distinguish such lesions from PNETs. Pancreatic resection is an effective choice to achieve long-term survival and ensure quality of life in most patients with PmRCCs. Further studies are needed to reveal the molecular mechanism underlying the biological behavior of the tumor.

## Abbreviations

PmRCC: Pancreatic metastatic renal cell carcinoma
RCC: Renal cell carcinoma
CT: Computed tomography
MRI: Magnetic resonance imaging
DP: Distal pancreatectomy
PD: Pancreaticoduodenectomy
DPPHR: Duodenum-preserving pancreatic head resection
PNET: Pancreatic neuroendocrine tumor
PET–CT: Positron emission tomography–CT
QOL: Quality of life
